# USEFULNESS OF REVISED SIMPLIFIED SHORT-TERM COGNITIVE SCREENING TEST (STMT-R) IN ACUTELY ILL GERIATRIC PATIENTS

**DOI:** 10.1093/geroni/igac059.2258

**Published:** 2022-12-20

**Authors:** Hiroshi Yamamoto, Kenichi Ogawa, Yasushi Hisamatsu, Tatsuya Ishitake

**Affiliations:** Yamamoto Memorial Hospital, Imari-city, Saga, Japan; Yamamoto Memorial Hospital, Imari-city, Saga, Japan; Yamamoto Memorial Hospital, Imari-city, Saga, Japan; Kurume University School of Medicine, Kurume-city, Fukuoka, Japan

## Abstract

**Background:**

Dementia can be a major cause of mortality and morbidity in geriatric patients. So, it would be essential to assess their mental state.Aims: We aim to appraise the impact of cognitive dysfunction on the long-term prognosis on STMT-R as a quicker and sensitive cognitive identification in acutely ill geriatric patients.

**Methods:**

The inclusion criteria were to measure geriatric patients by STMT-R at admission, age≧50yo and being non-critical ill. Between October 2014 and September 2015, 836 were enrolled (52.4% female, mean age: 78.9 years). STMT-R≦4 was considered as cognitive dysfunction. Following the collection of clinical data, survival was subsequently measured for 7-8 years until January 2022. Cox’s proportional hazards regression models were used to evaluate the hazard of death according to the dementia severity, with adjustment for potential covariates. Survival was estimated using Kaplan-Meier method.

**Results:**

Among enrolled subjects, 144 were unable to complete the test due to severe dementia (ITG). 433 had cognitive dysfunction (STMT-R≦4; CDG) and 259 didn’t have cognitive dysfunction (STMT-R>4; NCDG). The survival curves for death among three groups were significantly decreased in the CDG and ITG compared with the NCDG. The risks for mortality in the ITG and CDG are 3.92 (hazard ratio; 95% confidence interval:2.74-5.61, p< 0.001) and 1.82(1.33-2.51, p< 0.001) compared with the NCDG as reference.

**Conclusion:**

1) It was suggested that severity of cognitive dysfunction at admission has independently an impact on survival rate in acutely ill geriatric patients.2) STMT-R may also be useful for the future bedside or remote cognitive assessment.

